# Ideal timing of indwelling catheter removal after robot-assisted radical prostatectomy with a running barbed suture technique: a prospective analysis of 425 consecutive patients

**DOI:** 10.1007/s00345-019-03001-4

**Published:** 2019-11-14

**Authors:** Sebastian Lenart, Ingrid Berger, Judith Böhler, Reinhard Böhm, Georg Gutjahr, Nikolaus Hartig, Daniel Koller, Michael Lamche, Stephan Madersbacher, Michael Stolzlechner, Claudia Elisa Wayand, Anton Ponholzer

**Affiliations:** 1 Department of Urology and Andrology, St. John of God Hospital Vienna, Krankenhaus der Barmherzigen Brüder Wien, Johannes-von-Gott Platz 1, 1020 Vienna, Austria; 2 Department of Urology, Paracelsus Medical University, Salzburg, Austria; 3 Department of Mathematics, Center of Research in Analytics and Technologies for Education, Amrita Vishwa Vidyapeetham, Amritapuri, Kollam, India; 4 Department of Surgery, St. John of God Hospital Vienna, Krankenhaus der Barmherzigen Brüder Wien, Vienna, Austria; 5 Department of Surgery, Paracelsus Medical University, Salzburg, Austria; 6 Department of Urology, Kaiser Franz Josef I Spital, Vienna, Austria; 7 Sigmund Freud Private University, Vienna, Austria

**Keywords:** Catheter removal, Robot-assisted radical prostatectomy

## Abstract

**Objective:**

To compare prospectively early outcome and complications of catheter removal after robot-assisted radical prostatectomy (RARP) on the 4th or 7th day with a standardized running barbed suture technique.

**Introduction:**

The time point of removing the indwelling catheter after RARP mainly depends on institute’s/surgeon’s preferences. Removal should be late enough to avoid urinary leakage and complications such as acute urinary retention (AUR) but early enough to avoid unnecessary catheter indwelling.

**Materials and methods:**

A consecutive single-institutional series of patients underwent RARP between July 2015 and August 2017 and were entered in a prospectively maintained data base. Between July 2015 and December 2016 a cystogram was performed on 7th postoperative day (group A), thereafter the cystogram was performed on 4th postoperative day (group B). Incidence of acute urinary retention (AUR), urinary tract infections (UTI) and adverse events between the two cohorts was compared.

**Results:**

425 patients were analyzed (group A: *n* = 231; group B: *n* = 194). Both cohorts were comparable regarding demographic and oncological parameters. Watertight anastomosis was present in 84.8% in group A and in 82.5% in group B, respectively. AUR within 4 weeks after RARP occurred in 2.2% (*n* = 3) in A and 9.4% (*n* = 15) in B (*p* = 0.001). AUR within 72 h after catheter removal occurred in group A: 1% (*n* = 2) and in group B: 6.3% (*n* = 10) (*p* = 0.005). Symptomatic urinary tract infections occurred in 8.2% (*n* = 16) in group A and in 6.9% (*n* = 11) in group B. There were no differences in the rate of secondary anastomosis dehiscence. Age, BMI, prostate size, surgeon, or intraoperative bladder neck reconstruction were not correlated to the occurrence of AUR or UTI.

**Conclusions:**

The removal of indwelling catheter on day 4 after a RARP with a running barbed suture shows similar anastomosis leakage rates as on the 7th postoperative day. However, AUR rates are higher for early removal. Patients scheduled for early removal should be carefully informed about the increased risk for AUR. Catheter indwelling time does not represent a risk factor for UTI.

## Introduction

The ideal time of catheter removal after robot-assisted radical prostatectomy (RARP) is still controversially discussed. Timing of catheter removal shows a wide range, according to institute’s/surgeon’s preferences. Only a few studies analyzed the issue of timing of catheter removal in more detail with heterogeneous study designs, all showing that early removal is feasible, yet prone to a higher complication rate. Early catheter removal may result in a higher rate of re-catheterization due to acute urinary retention (AUR), secondary urinary leakage or anastomotic disruption [1, 2]. A prolonged catheterization time, however, leads to patient discomfort and implicates the risk of catheter dependent complications, such as urinary tract infections, and negatively impacts on short- and intermediate urinary continence rates [3–6]. AUR requires the need of re-catheterization and—potentially—re-hospitalization. Thus, determining the ideal timing for catheter removal enhances patient’s care in the postoperative setting.

The aim of this study was to compare prospectively sequelae of catheter removal on the 7th versus the 4th day after RARP in a consecutive series with a standardized anastomosis technique within the first 30 days after surgery.

## Materials and methods

### Patients

A prospectively maintained database was implemented in 2011 when RARP was introduced to our institution. Herein a consecutive series of patients, operated between July 2015 and July 2017, was analyzed. Between July 2015 and December 2016 a cystogram was performed on the 7th postoperative day (group A), thereafter the cystogram was performed on day 4 (group B) after surgery. A retrograde cystography was performed by instillation of 100 ml 50% cystografin during X-Ray fluoroscopy via the transurethral catheter. In absence of anastomosis leakage, the catheter was removed. In case of urinary leakage, the catheter remained in place until further cystogram showed no leakage any more. These patients were excluded from this study. In 183 (43.1%) patients an extended pelvic LND was performed. In 31 patients (7.3%) lymphatic node metastases were detected. Recruitment for this study was consecutive and prospective so patients with prior TURP or difficult anatomy of the prostate lobe were not excluded. Eight patients had a TURP prior to RARP.

All patients received perioperative antibiotic prophylaxis with single shot of 3 g Cefuroxim i.v. or a single shot of Fluorchinolones, in case of contraindication to Cephalosporines. All patients were informed to seek our outpatient clinic in case of any problems after discharge from the hospital, documented as long as 30 days after surgery. All patients underwent a follow-up visit 30 days after surgery with a urinary dipstick analysis and abdominal ultrasound.

Lymphatic drainage was inserted in all cases. The time of drainage removal depended on the drain output and drain was removed if drain volume was < 150 ml per day.

### Anastomosis technique

RARP was performed via conventional transperitoneal approach. First, seminal vesicles were prepared through a dorsal approach. Second, bladder was mobilized and preparation of the bladder-neck performed. After developing lateral surface of the prostate via nerve-sparing or non-nerve-sparing procedure, the dorsal vein complex was separated and the urethra was developed. The Plexus Santorini was ligated with a V-Lock™ suture. A modified Rocco-Stitch, with re-adherence of the urethra at 5 and 7 o’clock was performed. The bladder neck and the urethra were re-anastomosed 1:1 with a running barbed suture and re-adjusted to a fit anastomosis. The anastomotic technique was identical throughout the entire study period with an attempt of bladder neck preservation and the anastomosis done by a running barbed suture. The suture was a 2-armed Monocryl 3/0 and ran at the blunt urethra at 6 o’clock in both directions and was accomplished with 3 knots. A retrograde filling of the catheter with 100 ml NaCl (Sodiumchloride) was performed during surgery to prove the sealing of the anastomosis. If a leakage was noticed, further anastomosis stitches were performed. In all cases a Charrière 18 (18 French) silicone catheter was inserted.

### Outcome parameters and statistical analysis

Main outcome parameters were prevalence of a tight anastomosis, AUR within 3 days after catheter removal, AUR within 4 weeks after catheter removal and prevalence of UTI within 4 weeks after catheter removal. Statistical significant differences between the two treatment groups were assessed using Student’s *t* test, *χ*^*2*^-*test* for sufficient large case numbers and Fisher’s exact test for small case numbers. Additionally logRank-test was used to compare event times for AUR 72 h and AUR. Multivariate and univariate regression analyses were performed for detecting influence or risk factors for the occurring events.

## Results

### Patients

A total of 425 patients were analyzed (group A: *n* = 231; group B: *n* = 194). Descriptive analysis and demographics of the patient population are summarized in Table 1. RARP were performed by seven different surgeons with homogenous distribution in both study cohorts.

**Table 1:** Description of patient cohort (*n* = 425)

	*N*	Mean	CI	7th	4th	
				Mean		*p*
Age	425	65.19	64.52–66.01	64.59	64.73	0.855
Size (cm)	425	176.49	175.47–177.26	176.08	177.36	0.140
Weight (kg)	425	85.81	84.65–87.31	84.35	86.75	0.093
BMI	425	27.86	27.14–29.17	27.89	27.56	0.739
PSA value (ng/ml)	414	9.77	8.72–10.93	9.99	9.21	0.478
Specimen weight (g)	424	52.01	50.35–54.35	49.90	53.41	0.077

Final histology: Gleason Score		cT		pT				pN		
		Overall				7th	4th		7th	4th
6	114 (26.8%)	1b	16 (6.5%)	2a	36 (8.5%)	18	18	0	136	106
7 (3 + 4)	166 (39.1%)	1c	131 (53%)	2b	15 (3.5%)	10	5	1	17	14
7 (4 + 3)	74 (17.4%)	2a	47 (19%)	2c	279 (65.6%)	147	132	X	78	74
8	34 (7.9%)	2b	19 (7.7%)	3a	60 (14.1%)	39	21			
9	36 (8.5%)	2c	34 (13.8%)	3b	35 (8.2%)	17	18			
10	1 (0.2%)									

### VUA (vesicourethral anastomosis) leakage

Postoperative cystography showed no difference in sufficiency rates of the VUA between group A and group B. Tight anastomosis were noticed in 84.8% (group A) and 82.5% (group B). No differences in the rate of VUA leakages were found between localized and locally advanced stages neither in group A nor in group B. No differences of sufficiency rates of the VUA between different surgeons were found. Patients with leakage in the cystography were excluded of the follow-up since the indwelling catheter was left in place.

### Aur

All patients had a successful first spontaneous micturition after catheter removal. AUR within 72 h after catheter removal occurred more often in group B than in group A (A: 1%, *n* = 2; B: 6.3%, *n* = 10; *p* = 0.005). AUR within 4 weeks after surgery also occurred more often in group B than in group A (A: 1.5%, *n* = 3; B: 9.4%, *n* = 15; *p* = 0.001). Most cases of AUR occurred within 72 h after catheter removal. AUR occurred on 1st (7 ×), 2nd (3 ×), 3rd (2 ×), 4th (1 ×), 5th (1 ×), 7th (1 ×), 21st (1 ×) and 28th (2 ×) day after catheter removal, respectively (hazard ratio [HR] = 6.31; 95% CI = 1.83–21.81; *LogRank*-*Test p* = 0.001). AUR was treated with a reinsertion of a Charrière 18 (18 French) silicone catheter in all cases, carried out by urologists of our institute. These procedures were uncomplicated in all cases. No differences were noticed between the two groups. After AUR, catheter was left in place for additional 4–7 days. A control cystogram was not part of the protocol but was performed in seven cases consistently, showing watertight anastomosis. No case of secondary anastomosis dehiscence occurred. None of preoperative or intraoperative variables (age, height, weight, BMI, PSA value, clinical and pathological stage, prostate weight, surgeon, surgery time, resection margins, complications after surgery like lymphocele collection or bleeding) showed an association to the rate of AUR, neither in multivariate logistic regression nor backwards stepwise logistic regression (see Table 2). Four of the eight patients with prior TURP had a cystography on postoperative day 4 and four on postoperative day 7. None of these patients experienced AUR.

**Table 2:** Stepwise backward regression for variables

Removal of variable	1	2	3	4	5	6	7	8	9	10	11	12
Chi-Quadrat	− 0.002	− 0.004	− 0.003	− 0.078	− 0.404	− 0.169	− 0.132	− 3.339	− 2.012	− 1.889	− 1.99	− 3.849
*p* value	0.97	0.95	0.96	0.78	0.53	0.68	0.72	0.34	0.16	0.17	0.16	0.05

1: clinical stage, 2: age, 3: surgeon, 4: PSA-value, 5: BMI, 6: weight, 7: height, 8:, complications, 9: specimen weight, 10: pathological stage, 11: resection margins, 12: surgery time

### UTI (urinary tract infections)

UTI were defined as painful voiding and increased voiding frequency with positive dipstick analysis for leucocyte esterase and facultative positivity for nitrites within the 30 days of follow-up. There were no significant difference in the occurrence rates of symptomatic urinary tract infections between the two groups (A: 8.2%, *n* = 16; B: 6.9%, *n* = 11; *p* = 0.660) (Table 3).

**Table 3:** Results of catheter removal on 4th versus 7th day after RARP

	7th in % (*n*)	4th in % (*n*)	OR	*p*	CI
Sufficient anastomosis	84.8 (196)	82.5 (160)	1.184	0.521	0.707–1.984
AUR	1.5 (3)	9.4 (15)	6.66	0.001	1.891–23.419
AUR < 72 h	1 (2)	6.3 (10)	6.47	0.005	1.396–29.956
UTI	8.2 (16)	6.9 (11)	0.836	0.66	0.377–1.857

## Discussion

The principle goal of minimal invasive approaches is to minimize surgical complication rates and adverse events. To improve patient’s quality of life in the postoperative period, reduced catheter indwelling time is an objective. Compared to the open approach, RARP is associated with a lower transfusion rate and less postoperative pain combined with similar functional and oncological outcomes [7–9]. Despite its larger costs, RARP has largely replaced the open approach, where available [10, 11]. Improved visualization of the bladder neck and urethra during RARP led to decreased rates of VUA leakage as well as decreased rates of AUR, independent of time of catheter removal, compared to open surgery techniques [1, 2, 12–15].

**Fig. 1:**
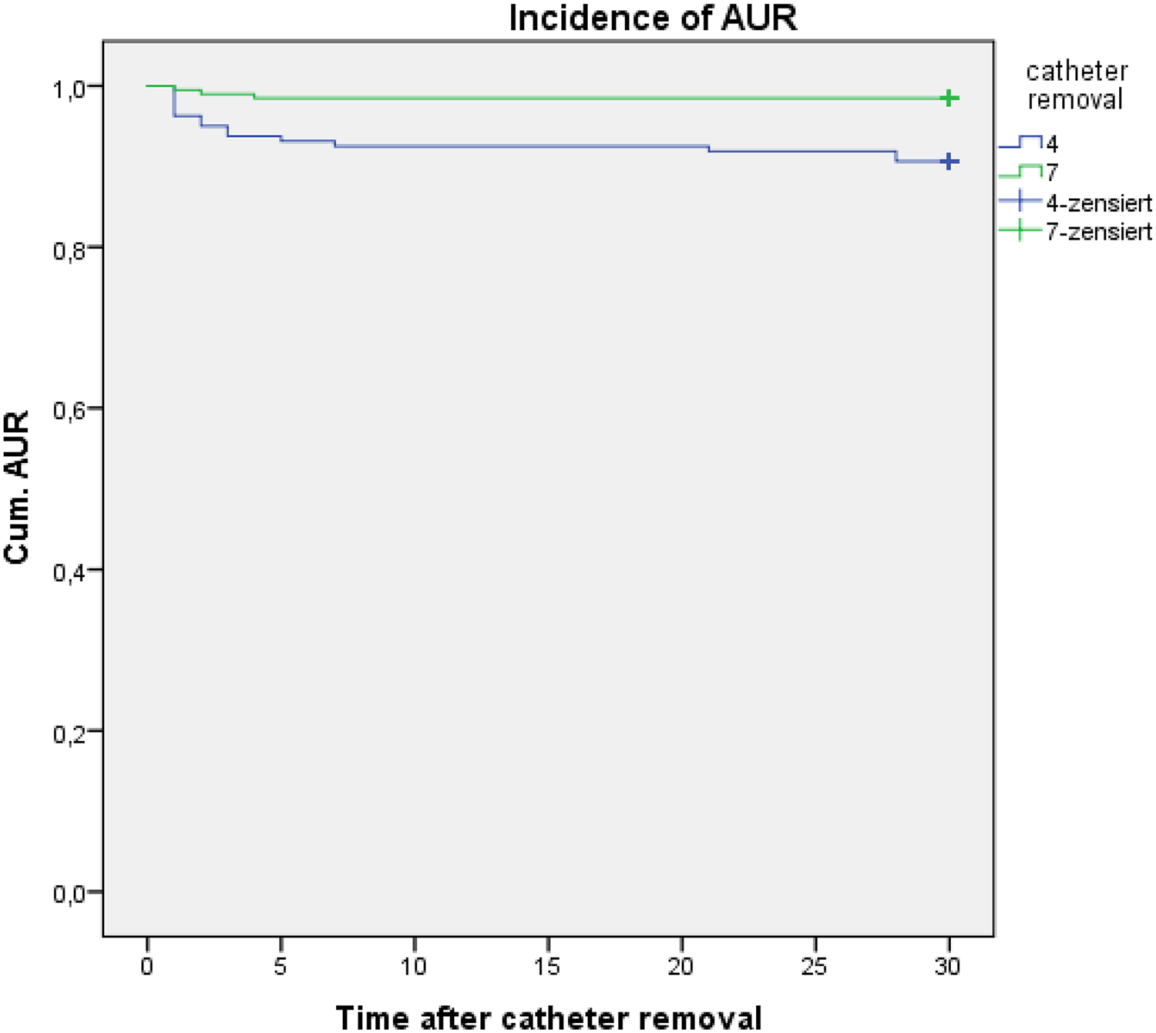
Cumulative incidence of AUR within 30 days after catheter removal on 4th or 7th postoperative day

The major finding of our prospective study was that early catheter removal after RARP is feasible yet associated with a higher AUR rate. To the best of our knowledge, no other prospective study with a standardized suture technique and a standardized anastomosis assessment, with this number of patients after RARP, is available. Most cases of AUR (67%) occurred within the first 3 days after catheter removal, showing that the first week after surgery is a crucial period for developing AUR (6.3% vs. 1%; *p* = 0.005) (Fig. 1). We could not detect any risk factor favoring the development of AUR, neither in univariate nor in multivariate analyses.

Despite its prospective design and a standardized suture/postoperative assessment, several limitations have to be taken into account. The major limitation is the lack of a randomized study design. VUA leakage rates were consistent between the two time points of catheter removal, but both series were consecutive—potentially biased by increased surgical experience. However, only patients without VUA leakage were followed up and included in further analysis, since in case of leakage the indwelling catheter was left in place. The decision for early/late catheter removal was not linked to a surgeon since the two groups were recruited one after another. All surgeons had a comparative experience in RARP procedures. Similar surgeons’ experiences are expressed in similar urinary leakage rates in both groups for all surgeons. AUR occurred more often in the group of early catheter removal, which was the later consecutive group. Thus, increased experience of all surgeons might had led to improved VUA leakage rates and decreased AUR rates. Thus, a prospective randomized trial between early and late catheter removal is necessary to diminish the impact of increased surgeon’s competence.

Second, although we could not identify any specific risk factors for developing AUR, several potential parameters, such as nerve sparing or preoperative voiding patterns were not routinely assessed. The impact of nerve-sparing procedure on improved functional outcomes, like early urinary continence, has already been shown in various trials [15].

Several studies have shown that shortening indwelling catheter time after RARP improves patient’s quality of life and reduces the length of hospitalization. Table 4 describes duration of catheterization of selected series suggesting that catheter removal is feasible as early as 2–4 days after RARP, with similar VUA leakage rates but a higher rate of AUR in most of the series [1, 15–18]. These observations are largely confirmed by our study. Herein, improved bladder neck sparing and less tissue handling might be the underlying causes for consistent leakage rates in early catheter removal. Bladder neck sparing is not always feasible, especially in locally advanced stages. However, AUR and VUA leakage rates were not higher in the group of > pT2 stages. Despite improved patient’s comfort due to decreased catheter indwelling time, higher rates of AUR need to be taken into account following early catheter removal [3]. As exemplified in Table 4, AUR rates range between 1.5 and 11% for catheter removal prior 3rd day after surgery, shown by almost all studies. All studies mentioned first spontaneously micturition after catheter removal. AUR occurred in most cases within 72 h after catheter removal.

**Table 4:** Overview of publications analysing AUR after LRP/RARP

Author	Study design	*N*	Surgery	Catheter removal	AUR % (*n*)	*p*	Suture
Lista et al. [16]	Prospective	153	RARP	3th 5th	1.4% (1/72) 1.4% (1/74)	0.9	Unknown
Alnazari et al.[2]	Retrospective	740	RARP	4th 7th	4.5% (16/351) 0.2% (1/389)	0.004	Running
Gratzke et al. [15]	Prospective	74	RARP	2th 6th	11% (4/37) 8% (3/37)	0.691	Aalst technique
Matsushima et al. [17]	Prospective	113	LRP	2th 4th	22.8% (13/57) 14.3% (8/56)	0.244	Running
Kheemes et al. [1]	Retrospective	1.026	RARP	3/4th 5/7th	5.8% (22/381) 0.5% (3/645)	0.0001	Running
Current study	Prospective	425	RARP	4th 7th	9.4% (15/160) 1.5% (3/196)	0.001	Running

The pathogenesis for AUR after prostatectomy is not clearly understood. Despite a considerable retention rate of 6.3% in our series, all patients were able to void spontaneously immediately after catheter removal. Some authors propose that AUR is due to edema of the VUA, an increased bladder neck muscle tone or postoperative pain [1, 2, 19, 20]. Patel et al. showed reduced AUR rates with Tamsulosin but the effect is likely to be dependent on the grade of bladder neck sparing [20]. RARP offers an improved setting for achieving bladder neck sparing, which would allow alpha-antagonists to mediate resistance relaxation. Further, different anastomosis procedures might contribute to developing perianastomosical edema. Anastomosis suture techniques and bladder neck sparing can contribute to decreased obstruction after catheter removal. If edema of the VUA is the reason for early AUR, preventive strategies to reduce edema should be evaluated. Since no risk factor could be identified in our analysis, further prospective assessment of these risk factors is therefore needed.

UTI are hardly ever mentioned in the early postoperative phase after RARP, although they undeniably put a strain on the patient’s well-being. Assessing relevant UTI needs to be defined since not all patients with positive leucocyte esterase in the urinary deep stick analysis need to be treated or experience hazard. Further, using prophylactic antibiotic therapy with ciprofloxacin for instance, did not show to be effective in the prevention of UTI after RARP [21]. However, there is no increased risk for UTI with increased catheter indwelling time.

## Conclusion

Removal of indwelling catheter on 4th day after a RARP shows similar anastomosis leakage on 7th day. However, AUR rates are higher on 4th day and need to be taken into account. Results from this study influenced our policy in catheter removal. Although the decision on the time point of removal still remains the surgeon’s decision, patients scheduled for 4th day removal are carefully informed about the increased risk for AUR.

Further randomized controlled studies with focus on the anastomosis procedure and strategies to reduce anastomotic edema, should be enrolled.
